# Facilitating Systems Thinking Through Arts-Based STEM Integration

**DOI:** 10.3389/feduc.2022.915333

**Published:** 2022-06-23

**Authors:** Robert William Danielson, Elizabeth Grace, Alison Joanne White, Molly Louise Kelton, Jeb P. Owen, Kristin Saba Fisher, AnaMaria Diaz Martinez, Maria Mozo

**Affiliations:** 1 Department of Kinesiology and Educational Psychology, College of Education, Washington State University, Spokane, WA, United States,; 2 Department of Teaching and Learning, College of Education, Washington State University, Spokane, WA, United States,; 3 4-H Youth Development Regional Specialist (Extension), College of Agricultural, Human, and Natural Resource Sciences, Washington State University, Yakima, WA, United States,; 4 Department of Teaching and Learning, College of Education, Washington State University, Pullman, WA, United States,; 5 Department of Entomology, College of Agricultural, Human, and Natural Resource Sciences, Washington State University, Pullman, WA, United States,; 6 Department of Teaching and Learning, College of Education, Washington State University, Pullman, WA, United States,; 7 Human and Family Development Regional Specialist (Extension), College of Agricultural, Human, and Natural Resource Sciences, Washington State University, Pasco, WA, United States,; 8 Biotechnology, MiraCosta College, Oceanside, CA, United States

**Keywords:** afterschool education, science/arts collaboration, STEM/STEAM, photography, illustrations, K-12

## Abstract

Engaging with socio-scientific issues often involves making sense of how – and for whom – actions, choices, and policies might affect aspects of daily life. Understanding the complexity of socio-scientific issues also requires recognizing the interconnectedness of – and working across – multiple communities and professions. We suggest that art, whether musical composition, illustrations, or sculpture / collage across materials would promote the synthesis of different types of knowledge across different scales and systems. The present investigation seeks to understand how arts integration into STEM curriculum could support systems thinking around socio-scientific issues, specifically around the issue of pathogen transmission in rural-agricultural communities. Our after-school program, which works with 3rd – 5th grade students in rural-agricultural communities, leverages the arts to promote systems-level understanding of zoonotic diseases and ecosystem dynamics. A total of 23 students across two sites located in rural communities in the Western United States participated in our afterschool program. We found that after completing the program students expanded their understanding of both the connections between concepts and an understanding of careers related to ecosystem dynamics. We suggest that educators can integrate both arts and sciences together to enhance systems thinking and expand student perception of the interconnectedness of STEM disciplines and their everyday lives.

## INTRODUCTION

Socio-scientific issues (e.g., climate change, global pandemics, or vaccination policies) require citizens to be both educated and engaged with the science and politics of these situations to meaningfully participate in society and their communities. Engaging with socio-scientific issues often involves making sense of how – and for whom – actions, choices, and policies might affect aspects of daily life. In many cases, relatively complex causal webs can lead to unexpected consequences. For example, the BBC (Reality Check Team, 2021) recently ran a story about some of the surprise outcomes of Brexit (e.g., lorry drivers had their ham sandwiches confiscated at the Dutch border) and this may lead individuals to express regret, frustration, or general dissatisfaction with unintended outcomes (McGee, 2021). While sandwiches may seem inconsequential, health-science researchers have predicted that Brexit-related changes to food import and export could significantly affect public health outcomes like cardiovascular disease over the long run (e.g., Seferidi et al., 2019).

Understanding the complexity of socio-scientific issues also requires recognizing the interconnectedness of – and working across – multiple communities and professions. For example, understanding and predicting connections across public health, Brexit, and border regulations calls for interdisciplinary conversations across trade economists, nutritional scientists, statistical modelers, and transportation industry workers, to name a few. While we (humans) have some control over these events (e.g., setting restrictions on food imports), other events are less clear. For example, while the global economic recession in 2006–2007 caused many in California to lose their homes, the massive surge of foreclosed homes led to a significant increase in neglected swimming pools. These swimming pools, in turn, became excellent breeding ground for mosquitoes, which led to a massive mosquito boom, and a nearly 275% increase of West Nile Virus cases in humans (Reisen et al., 2008). While the connection between global economic downturns and local mosquito-borne disease may seem disconnected, an interdisciplinary systems approach would illuminate these connections. Yet interdisciplinarity is often underemphasized in learning environments, especially school systems.

One reason foreseeing these consequences can be so difficult is because issues like Brexit are often representative of a complex system in which a change in one part of the system (restricting imports) will result in changes to other parts of the system (a lack of healthy food options that may impact public health). Understanding socio-scientific issues and their implications requires thinking that can integrate social, economic, environmental, and ethical dimensions of a large, interdependent system. Educational researchers conceptualize this through the lens of “systems thinking” – reasoning about the components of a system and the interactions among them (Hmelo-Silver and Azevedo, 2006). As the world becomes both more complex and more interconnected, a systems thinking approach to instruction benefits students.

In many cases, formal academic disciplines are taught as distinct, independent areas of study. However, to deeply understand the science around a global pandemic, for example, students would need to combine multiple disciplines (biology, medicine, economics, psychology, political science, etc.) and the components of these disciplines (the nature of science or science communication). Since the early 2000s, many countries have moved toward a more integrated approach to combine these disciplines to better prepare their students to address global issues. As STEM programs aim to train students with the skills required to respond and communicate about these global issues (Treagust and Tsui, 2014), systems thinking becomes a bridge to STEM literacy for all students.

The present investigation seeks to understand how arts integration into STEM curriculum could support systems thinking around socio-scientific issues, specifically around the issue of pathogen transmission in rural-agricultural communities. Our after-school program works with 3rd – 5th grade students in rural-agricultural communities and leverages the arts to promote systems-level understanding of zoonotic diseases and ecosystem dynamics. In what follows, we briefly review the literature regarding STEM education, arts integration, and systems thinking. Then we describe the current investigation and our attempts to both foster and measure systems thinking. Finally, we present findings that our arts-integration was successful at increasing understanding of both art techniques (as illustrated by the evolution of artistic expression) and systems thinking (as illustrated by an expansion of multiple systemic elements and their combination). We close by illustrating areas where future studies could improve upon our methodology.

## LITERATURE REVIEW

A major goal of STEM education has been to produce creative, diverse, and innovative problem solvers that will bring complex knowledge and skills to the evolving workforce (White, 2014; U.S. Department of Education, 2021). We use STEM in line with previous descriptions of STEM education that focus on integrating at least two STEM disciplines (for example, integrating mathematics and engineering to calculate the savings from newly designed weather insulation) to apply STEM concepts and attempt to solve real-world problems (Bybee, 2010; Honey et al., 2014, 2020; Vasquez, 2014). Many of the global industries that maintain our way of living, such as natural resource refineries, construction, and healthcare, depend increasingly on STEM integration and development (Gillespie, 2021). Providing students with a strongly integrated STEM framework can create more informed adults, who will be able to comprehend and participate in political and social conversations that have lasting global effects (i.e., the current impacts of COVID-19 misinformation on pathogen transmission).

Major issues surrounding STEM programs are the culture and traditional practices around STEM teaching and learning that can be perceived as exclusionary (Tan and Barton, 2018). While the goal is typically to train all students with STEM skills, many students feel disconnected or that these programs are not open to them, leading to disengagement or dropout. To increase participation, some authors have called on including art curriculum within STEM programs and curricula (Ainsworth et al., 2011; Forbus and Ainsworth, 2017). STEAM allows students to interact with and create multiple models of complex ideas (Allina, 2018; Lima and Timm-Bottos, 2018). Incorporating art may increase access points for students to engage in STEM by creating environments that are inclusive of and attractive to a greater diversity of learners. Expressing ideas and new learning through art can support equitable environments for students with a wide range of prior knowledge, language barriers, cognitive diversity, or learning disabilities (Schwartz, 2019). Arts integration can increase recruitment and retention of female students within STEM programs, working to increase gender equality within STEM pathways (Wajngurt and Sloan, 2019). Leveraging the arts may also boost inclusion and engagement by allowing students’ lived experiences to be recognized and validated as experiences from their daily lives and their communities can be applied to express and explore STEM, and to engage in their creativity and imaginations (Ngss Lead States, 2013; Calabrese Barton and Tan, 2018).

Young children, many of whom do not matriculate into more advanced STEM courses (Garrison, 2013), may begin to develop an early interest in STEM practices when viewed through the lens of art, imagination, and creative expression. It also allows for a deeper investigation of scientific practices from an artistic vantage point – as Bob Ross was fond of saying, there are no mistakes, just happy accidents. Including arts-based approaches and coupling these approaches to science may function to maintain or increase students’ interest in both arts and science, connect learners to socio-scientific issues, and promote scientific epistemic practices (Ngss Lead States, 2013).

### Integrating Art Into STEM

Ainsworth and colleagues (2011) suggest five reasons for integrating art into science: (1) to enhance engagement, (2) to learn to represent in science, (3) to reason in science, (4) as a learning strategy, and (5) to communicate. Peppler and Glosson (2013) suggest that projects with open-ended tools contribute to deeper understanding of STEM concepts. We suggest that art can promote the synthesis of different types of knowledge across different scales and systems. Ngss Lead States (2013) highlights the “…influence of engineering, technology, and science on society and the natural world” (p. 1). This can often be seen as a topic for many contemporary art installations, showcasing the “…crosscutting concepts of patterns; cause and effect; scale, proportion, and quantity” (p. 1).

Most research has examined how art can be *leveraged* in service of teaching science, but one might ask, “what’s in it for the arts?”. For example, while Peppler and Glosson (2013) indicate how stitching is both a visually concrete and physically tangible way for students to engage with electrical circuits, they do not examine to what extent students learned about textile art for its own sake. Said another way, art has the ability not just to communicate science content to students, but additionally, allows students to communicate science *through* art, demonstrating both the emotional and intellectual aspects of science (Pelto, 2015). We appreciate these artistic connections with science and would like to push researchers and practitioners to use these opportunities to advance both disciplines. For example, the photograph of the “bathtub ring” around Lake Mead with a boat for scale tells a particular story and experience of rapid change that requires learners to construct and participate in (for example, see Ethan Mille /http://www.gettyimages.com/detail/452138628). However, we can also dig deeper into *what aspects* of this photograph make it so powerful, opening a discussion around composition and other photographic techniques. Practitioners can then transition into activities where students recreate this photo in their own community, illustrating both the change in their environment while also incorporating newly learned photographic techniques. Creating art can also leverage the meaning behind the numbers (see Jill Pelto’s gallery, https://www.jillpelto.com/gallery) where the trend line for the increase in wildfires evokes a visceral reaction in the viewer.

In line with Mejias et al. (2021), we sought to position art and science as “mutually instrumental” in our program design, addressing and valuing equally both arts and science learning goals and practices. Not only are both promoted in the project (advancing understanding of both the science content and artistic approaches) but also are intertwined. As described and exemplified in the Materials and Methods section, our main strategies for working toward this goal in the context of the current study have been (a) identifying and designing for both arts and science learning objectives and (b) seeking specific synergies between scientific concepts and arts modalities and practices. Our approach also aims to connect science and art to the local community. For example, when students create photographs of the areas in their community that support mosquito habitats, they not only combine arts and science, but also connect and take ownership over their own community and highlight, at the local scale, some of the injustices present therein (Tan and Barton, 2018). STEAM education has also been noted as an opening to engage students in conversations of culture, equity, and social and environmental justice as they relate to STEM (Sochacka et al., 2016; Allen-Handy et al., 2021; Upadhyay et al., 2021). Integrating culturally specific art forms may offer affirmation of underrepresented students’ cultural aesthetic in STEM (Hunter-Doniger et al., 2018; Allen-Handy et al., 2021) and public displays create opportunity for community engagement in STEM issues and systems thinking (Guyotte et al., 2014; Sochacka et al., 2016) (For example, see the work of Isaac Cordal, http://cementeclipses.com/Works/follow-the-leaders/).

These public displays could play a role in the public synthesizing and understanding how ideas displayed today could have impacts felt within and across the community as we conceptualize and re-imagine how to live in times of rapid change. Yet, as mentioned above, few programs focus on the transdisciplinary nature of STEAM and potential increases in systems thinking. In the present investigation, we are interested in how arts integration in an afterschool program might support systems thinking about complex socio-scientific issues.

### Promoting Systems Thinking Through STEAM

Some scholars describe STEAM education as a transdisciplinary approach (Marshall, 2010; Guyotte et al., 2014; Peppler and Wohlwend, 2018) – “one that goes beyond, or transcends, the boundaries of particular disciplines” to “inform deliberation on a problem that is relevant to a real-world context” (Costantino, 2018, p. 102). Systems thinking is an example of a transdisciplinary benefit of STEAM education (Liao et al., 2016; Lima and Timm-Bottos, 2018), as art may encourage students to see relationships and adopt multiple perspectives – skills that not only support STEM outcomes but also broader capacities such as creativity, empathy, and critical thinking. In other words, transdisciplinary benefits such as systems thinking point to the potential of STEAM education beyond solely serving STEM to tap into the capacity of the arts to support cross-disciplinary, real-world problem solving.

Systems thinking is the ability to reason about complex systems. Complex systems can be defined as “interconnected components whose behavior is not explained exclusively by the properties of their components, rather the behavior *emerges* from the interconnectedness of the components” (Sabelli, 2006, p. 6). Although people engage with complex systems throughout their daily lives (e.g., traffic jams, flocks of birds), they often view such phenomena in isolation, through a simplified lens of linear cause and effect (Resnick, 1996; Wilensky and Resnick, 1999). Complex systems thinking has been identified as a critical cognitive skill for the 21st century (American Association for the Advancement of Science, 1993; Ngss Lead States, 2013).

Systems thinking has been identified as an underdeveloped skill in both adults and children (Hmelo-Silver et al., 2007). This has led scholars to emphasize the need for systems thinking learning and research in the elementary grades (Jacobson and Wilenksy, 2006; Assaraf and Orion, 2010; Danish et al., 2011; Danish, 2014; Hokayem and Gotwals, 2016), before children adopt linear ideas about relationships in systems (Forrester, 2007). Arnold and Wade (2015) point to Richmond (1994), defining systems thinking as the art and science of developing an increasingly deep understanding of the underlying structure of concepts and their interrelatedness. For example, as children see relationships and interactions across times, places, and disciplines, they expand their perception more broadly across the entire system (National Academies of Sciences, Engineering, and Medicine, 2022). Rather than adopting a single discipline’s approach to seeing a concept (e.g., transmission of airborne pathogens), adopting a systems approach allows students to expand their perception to include pathogen transmission as well as ways we can hinder or accelerate this transmission, and the broader impact of these polices on society writ large.

Unfortunately, systems-level thinking is not often developed well in schools. The end result of the fact-driven and superficial education process that many students engage with means that many students have difficulty understanding and interpreting complex and emergent systems (Penner, 2000; Chi, 2005; Hmelo-Silver and Azevedo, 2006; Jacobson and Wilenksy, 2006). This isn’t a developmental issue, however, as elementary-level children are capable of understanding complex systems when given the opportunity (Danish et al., 2011). We propose that very young children can and do understand how complex systems are related to one another, but may require more concrete connections to flesh these out. We suspect that arts-integration allows for the physical manifestation of these abstract connections, where students can create the links or draw the connections themselves. STEAM education may support systems thinking by allowing students to use art to integrate their personal experiences that exist within larger systems, offering them a context for exploring and discussing complex systems through the discussion of each other’s art (Guyotte et al., 2014).

The present investigation examines how participants develop their systems thinking, as evidenced by their capacity to (1) make distinctions across scales of analysis, (2) attend to multiple elements and their inter-relatedness, (3) understand underlying functions and structures, (4) recognize multiple interacting causal forces, (5) model and make sense of the cascading influences that changes in one part of a system can have on other system components, and (6) shift toward more decentralized forms of sense-making about scientific processes (e.g., Resnick, 1996; Penner, 2000; Hmelo-Silver and Azevedo, 2006; Sabelli, 2006).

## RESEARCH DESIGN

Our broader project, Health Education through Arts-based Learning (HEAL), is a partnership between university researchers and community organizations with an aim to ultimately increase rural Latino/Latina representation in biomedical fields and address disparities in STEM education, career development, and health outcomes for rural, predominately Hispanic communities by (1) building biomedical science interest, understanding, and systems thinking among young children and their communities, and (2) increasing rural STEM education capacity, both formal and informal, by leveraging the arts within curricula and evaluation about locally relevant disease systems (*e.g., West Nile Virus, enteric bacteria, COVID-19, Toxoplasmosis*).

The afterschool program of focus in this study was created by a team of interdisciplinary scholars (education, human development, public health communication, medicine, and biology) conducting iterative design-based research on STEAM programs related to health sciences through the lens of mosquito-borne pathogen transmission. We worked alongside upper elementary students (ages 8–12) to engage in both scientific investigations and studio activities to blend scientific and artistic understanding. We incorporated scientific illustration, cartography, photography, infographics, and collage creations to explore mosquito morphology, life cycle, and habitats as they relate to West Nile Virus (WNV) transmission. WNV is particularly relevant to these communities since they are disproportionately affected by the virus, which could be due to several factors including intersections of race/ethnicity, socioeconomic conditions, access to and use of testing (Silk et al., 2010; Agency for Healthcare Research and Quality, 2020), high-prevalence of agricultural employment (Meseck, 2020), and local landscapes with ideal conditions for the mosquitoes and birds that support WNV (Crowder et al., 2013).

We examine a subset of this artwork (the image selections and illustrations) in the present analysis, and its connection to related careers. Our current research question is as follows: “How did students’ systems thinking, as measured by student artwork, short answer responses, an image selection task, and a career selection task, change as a result of participation in our afterschool program?” A total of 29 students participated in our program, with varying attendance. Given the nature of after school program attendance, not all students attended all sessions, and as such, our numbers fluctuate across measures. However, by triangulating across multiple measures, we believe we paint an accurate account of the students’ learning that took place.

## MATERIALS AND METHODS

### Buzzing for Blood

Our instructional module, “Buzzing for Blood,” took place in two rural, predominantly Hispanic communities in (Washington State for review) State, where students attended two 90-min sessions per week for six weeks (18 total hours) in out-of-school community spaces (i.e., library classroom in Location B, housing complex community center in Location M). The experience culminated with youth art exhibitions where students shared their STEM-related artwork with families and community members. A typical afterschool experience would entail a check in / arrival period, snacks, warm-up illustrations or photography, and the content of the lesson. Designed to be both interactive and engaging, students often spent time creating their own art or taking pictures of the content as it could be found around their local community. This process was designed to allow for students to actively blend both science and art together at the same time, rather than separate these approaches.

### Curriculum

Table 1 provides an overview of each week-long unit. The program integrates explorations of mosquito morphology and ecology with multiple arts modalities: scientific illustration, infographics, photography, and cartographic arts. Arts modalities and topics were paired based on conjectured art-science synergies, for example between spatial ecology (e.g., habitat locations) and cartographic methods for representing neighborhoods and landscapes (Table 1, Unit 5). Our pedagogical approach aimed to equally address – and value – learning about specific arts modalities and mosquito science. Each week-long unit included programming focused on arts skills (e.g., shading techniques, Unit 1) as well as arts-based scientific experimentation and inquiry (e.g., iteratively observing and illustrating mosquito anatomy, Unit 1). Throughout the program we aimed to position students as scientific communicators using arts-based techniques to teach and engage with their communities, culminating in community-based art shows in which students displayed and discussed their scientific artwork with their families and community members.

### Participants

A total of twenty-nine students participated across both locations in some fashion. While the curriculum was designed for students ranging from grade 3–5 (ages 8–12), due to the location (within walking distance of students’ houses) and time (after school) many students would bring their younger siblings to participate as well. We did not collect data for children outside of those officially enrolled in the program, although we did invite these children to stay and engage with the activities. As such, their data is not presented here. Additionally, many students would miss one or more modules, some entering the program after it had started and others leaving the program before it had completed. We strived to include as many students as we could throughout the program, and therefore some of our numbers are inconsistent across measures. For example, fewer students completed the pre-program written assessment, but later joined the program and completed the post-program assessment. At site B, the 2010 census demographics indicate that approximately 88% of the ~1,000 residents identify as Hispanic / Latino. A total of 6 students participated at this location. Four of those completed demographic questionnaires, and of these four students, 3 were in 3^rd^ grade (the fourth was in 5^th^) and 3 indicated speaking both English and Spanish at home (with the fourth only speaking Spanish at home). At site M, the 2010 census demographics indicate that approximately 92% of the ~2,300 residents identify as Hispanic / Latino. A total of 23 students participated in this program but only 6 completed the demographic questionnaire. Of those 6, 3 were in 4^th^ grade and 3 were in 5^th^ grade, and 2 indicated speaking both English and Spanish at home (with four only speaking Spanish at home). We outline the number of students per analysis below.

### Measures

The present study examines systems thinking through four main assessments: an image selection task, a career selection task, a written assessment, and student illustrations.

The image selection task consisted of 36 images with varying levels of direct relevance to mosquitos and disease systems. Example images include: children (a food source for mosquitoes that students may have experienced), stagnant pools and horses (familiar sights to students but they may not recognize as direct food sources for mosquitoes), ambulances and medical professionals (direct consequences of disease), and money (indirect, abstract consequence of disease). This assessment was given at the beginning and end of the program. A total of 20 students completed at least one, but only 6 students completed both.

The career selection task consisted of 16 careers with varying levels of direct relevance to mosquitos and disease systems. Example careers include: pest control worker, doctor, and nurse (careers directly involved with mosquitos and disease and that students may have experience with), biologists, epidemiologists, and veterinarians (careers involved with mosquitos and disease that students may not have experience with). This assessment was given at the beginning and end of the program. A total of 19 students completed at least one, but only 6 students completed both.

The written assessment was comprised of 6 questions about mosquitos and mosquito habitats. In the present investigation, we examine 2 of these items, “why do you think mosquitos bite people?” and “what do mosquitos need to eat in order to live?”. This assessment was given at the beginning and end of the program. A total of 19 students completed at least one, but only 6 students completed both.

Prior to and immediately following the program, students completed the image selection and career selection tasks where they were instructed to circle images and careers related to mosquitoes and to write descriptions (on the images) explaining their selections. Mixed methods were employed to analyze data. For the present analysis, selected careers along with the selected images and written descriptions were coded in parallel with the six capacities of systems thinking noted above. Given the small number of students and the exploratory nature of data analysis, we present descriptive statistics and triangulate these results with students’ responses and explanations.

## RESULTS

Our findings reveal that student thinking around mosquitoes and disease systems greatly expanded after completion of the program, particularly through greater attendance to multiple elements and their relatedness and recognizing multiple interacting causal forces. We discuss these in isolation before triangulating findings across modalities.

### Image Selection

Examining all the data available, we find that the average number of images selected per student increased between the pre-assessment (*N* = 16, *M* = 7.4, SD = 3.9) and the post-assessment (*N* = 10, *M* = 21.8, SD = 5.0). The average number of careers selected increased between the pre-assessment (*N* = 12, *M* = 2.8, SD = 3.7) and (*N* = 12, *M* = 6.6, SD = 2.7) post-assessment as well. We interpreted that a greater selection of images and careers that pertained to mosquitos indicates that students were attending to an increased number of elements and their relatedness to mosquitoes after the program. This simple measure of volume additionally suggests an increased recognition of multiple interacting causal forces influencing mosquito systems.

**FIGURE 1 |:**
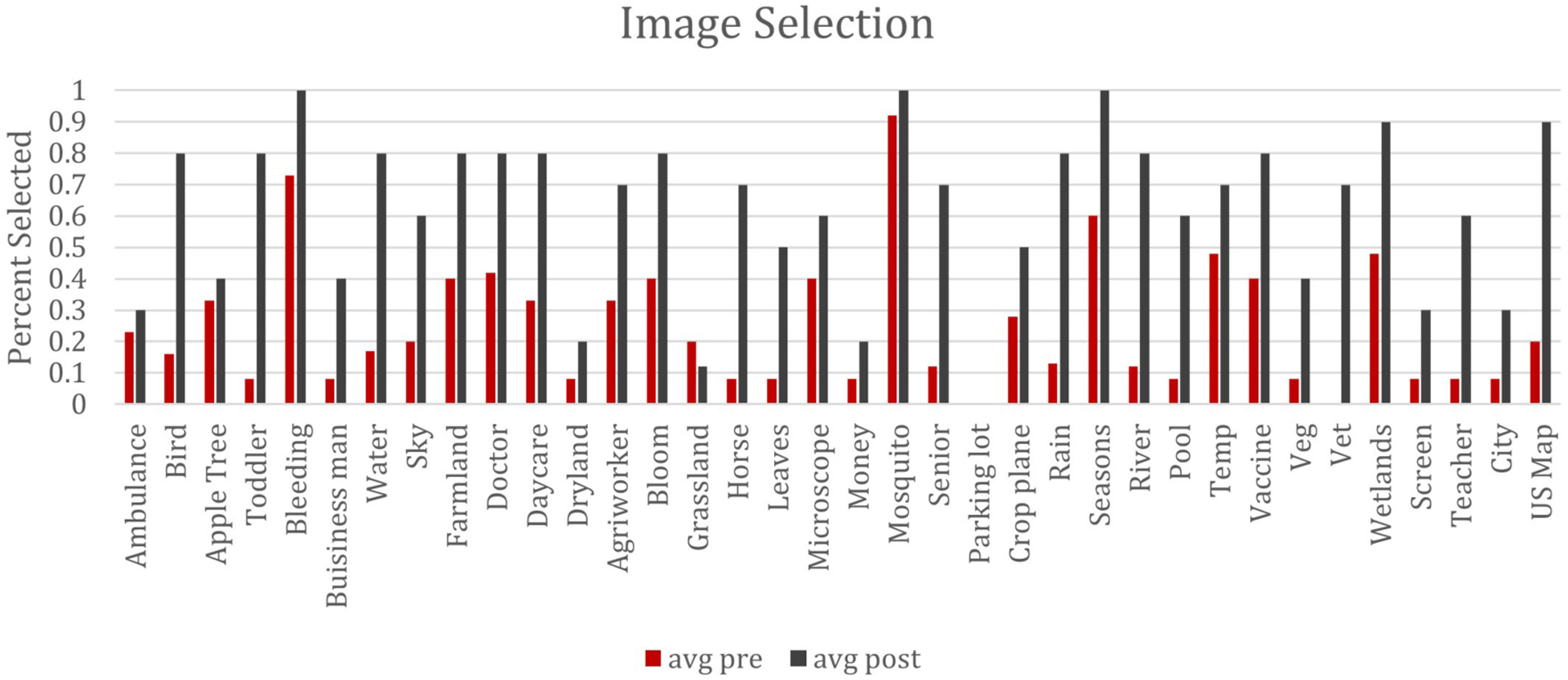
Percentage of images selected pre and post.

In addition to overall volume, several patterns emerged as to which specific images and careers were selected more often after the program. For example, while some images were consistently selected (humans, mosquitoes, blood), others were selected more often only in the post assessment (horses, birds, leaves, flowers), despite their direct relationship with mosquitoes (these all represent sources of food). Specifically, horses and birds were selected by less than 20% of the students before the program, but over 70% of the students selected them after the program. Leaves and flowers each saw an increase in selection rates of approximately 40%, which we interpret as students recognizing the broader scope of the sources of food for mosquitos and how their local community can support mosquito growth. Some of the images with the largest increase in selection included images related to mosquito habitats – stagnant water (60% increase), pools (50% increase), rain (60% increase), and a map of the United States (70% increase). Taken together, we see these increases as one way to measure how students are making connections across layers of a system, illustrating an increase in both absolute size and the level of connections. The overall patterns can be seen in Figure 1 below.

### Career Selection

**FIGURE 2 |:**
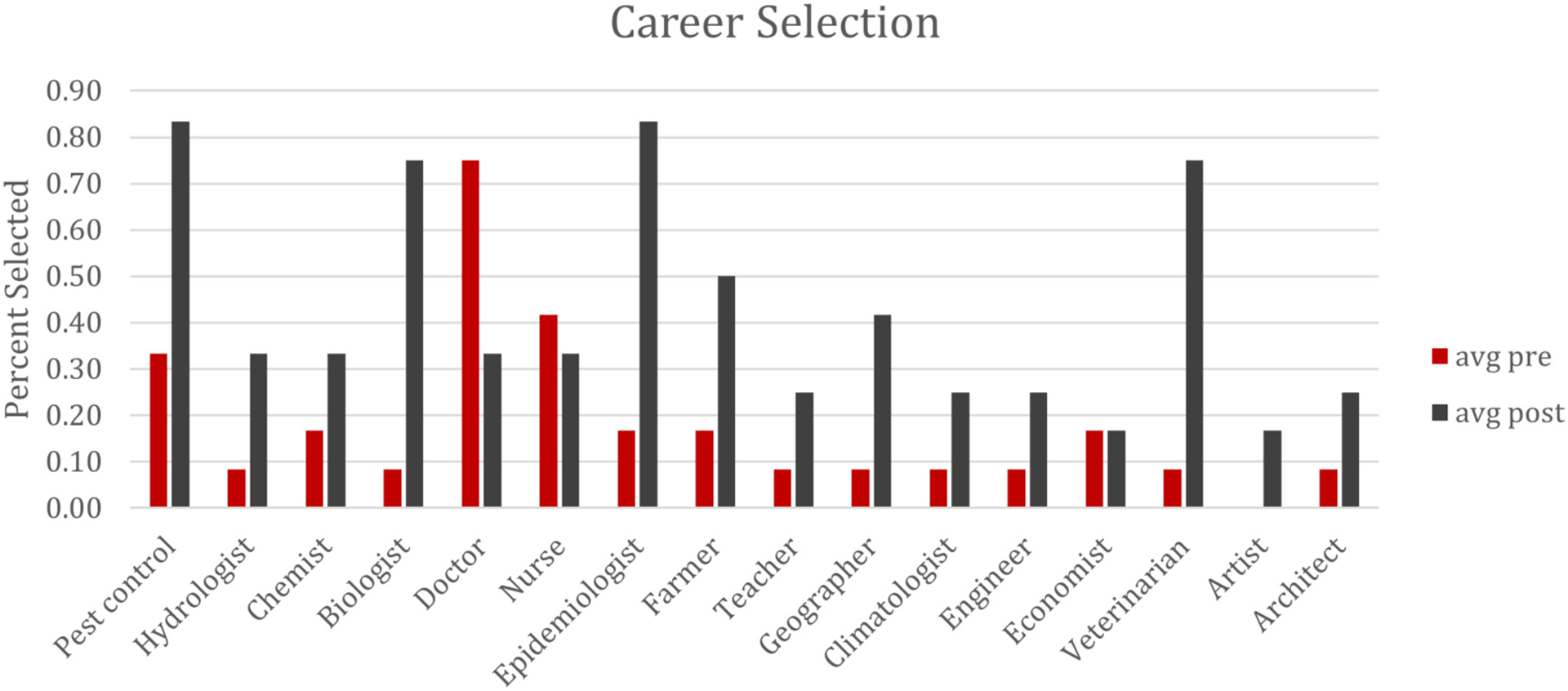
Percentage of careers selected both pre and post.

The increase in images selected was mirrored with an increase in career selections as well. First, one notable pattern emerges as to the low rate of selection of careers. Doctor (75%) and Nurse (40%) were by far the most selected in the pre-assessment, with Pest Control Worker (33%) selected as a close third place. We interpret these results to suggest that most students were not aware of what many of these careers do – but when asked which careers help with keeping people safe from mosquitoes, doctors, nurses, and pest control workers were an obvious choice based on student prior knowledge. What we find interesting is both the increase in selection of careers directly related to mosquitoes – Biologist (60%), Epidemiologist (60%), Pest Control Worker (50%), and Veterinarian (60%) – but also the depreciation of other careers directly related to health (Doctor ~50% and Nurse ~20%). Finally, there were large increases in the selection of other professions that deal with both the landscape and education (Hydrologist, Climatologist, Geographer, Farmer, and Teacher). We interpret these findings as evidence that students are expanding their understanding of both health- and science-related careers and making the connections between these terms and the careers they represent. The overall patterns can be seen in Figure 2 below.

As the images began to convey more complex and nuanced connections – for example, the image depicting money was designed to convey the cost of healthcare and disease mitigation both on the individual and community – students may have struggled to incorporate these into their understandings. Some images, including the ambulance and money, were selected by less than 30% of students at both pre and post assessment. Other images that we thought would be directly related to protection from mosquitoes – including window screens (10% selection rate at pre) and crop-dusting planes (33% selection rate at pre) only showed modest increases (approximately 20% each).

Our results also indicated a disconnect between both the image selection and career selection. For example, while the career of “Pest Control Worker” saw a 50% selection increase (from 33 to 83%), the crop-dusting airplane only increased from 33 to 53%. Another interesting trend was a sharp decline in students selecting more general concepts like the Nurse or Doctor careers (42% to 25% reduction for nurse, 75% to 25% reduction for doctor) coupled with a sharp increase in selecting more specialized careers like the capitalize). This may indicate that while some students are making these broader connections between constructs, there is still significant room to grow.

### Image Descriptions

Student descriptions further indicated changes in systems thinking. This feature of the image selection task allowed researchers to understand why students selected specific images. In addition to increased recognition of related elements and multiple interacting causal forces, student descriptions also revealed the capacity to make sense of the influence that changes in one part of a system has on other parts of the system. For example, images depicting temperature and seasons were selected more frequently in the post-assessment. These selections were accompanied with descriptions such as on “warm days they get out and bite people” (student data) and “in different seasons they leave and return” (student data). These data suggest that students are connecting the influence of larger systems such as weather and seasons on the behavior of mosquitoes. Another student was able to apply systems thinking when presented with the image of vegetables, relating it to their mother’s job as an agricultural worker and the presence of mosquitos in that environment. This connection created a foundation to build upon associating a higher risk of mosquito bites with the agricultural workforce.

### Short Answers

While only 6 students completed the short answer questions both pre and post, their statements offer insight into their understanding. Two questions were asked, “why do you think mosquitos bite people?” and “what do mosquitos need to eat in order to live?”. Regarding the first, only 4 (66%) students indicated that mosquitos bite people to drink their blood, with only 1 (17%) student indicating that they need the blood to lay their eggs. At the end of the program, all students understood that mosquitos bite people to drink their blood, with 4 (66%) understanding that the protein in the blood was required to lay eggs. Regarding the second question, 4 (66%) students knew that mosquitos eat blood, with the other 2 (33%) not sure what they eat. At the end of the program, all students understood that mosquitos drink their blood, with 3 (50%) further understanding that adult mosquitoes also drink nectar and larval mosquitoes eat decomposing leaves.

### Student Artwork

**FIGURE 3 |:**
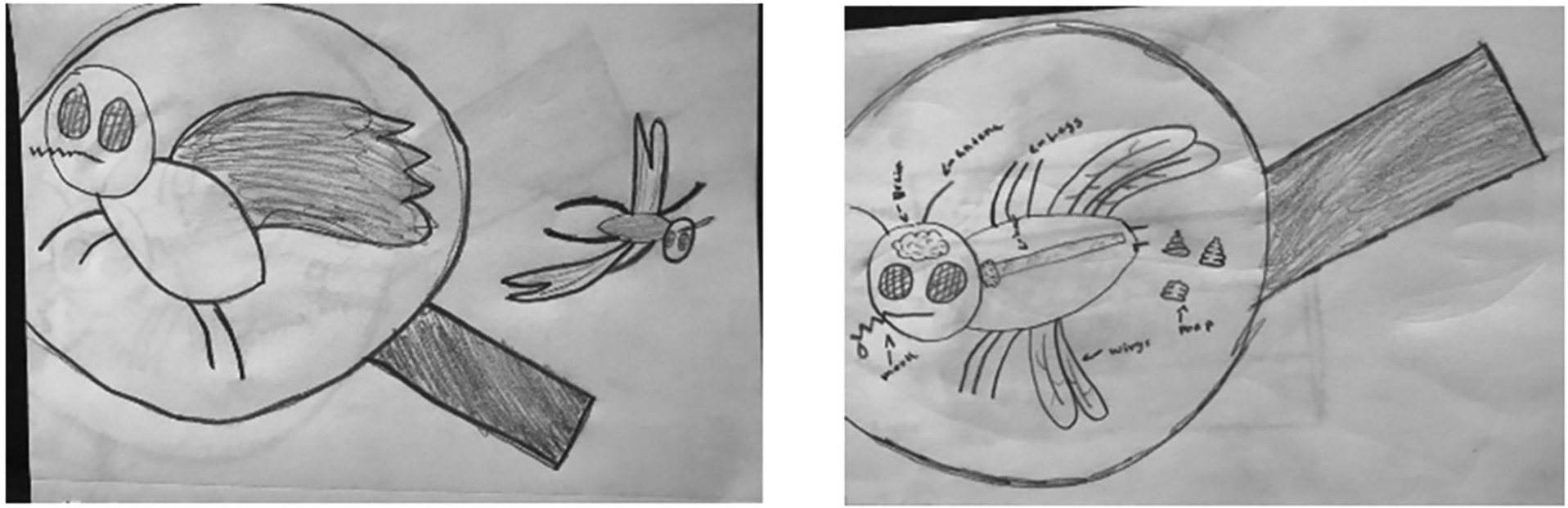
Student illustration of a mosquito under a magnifying glass.

**FIGURE 4 |:**
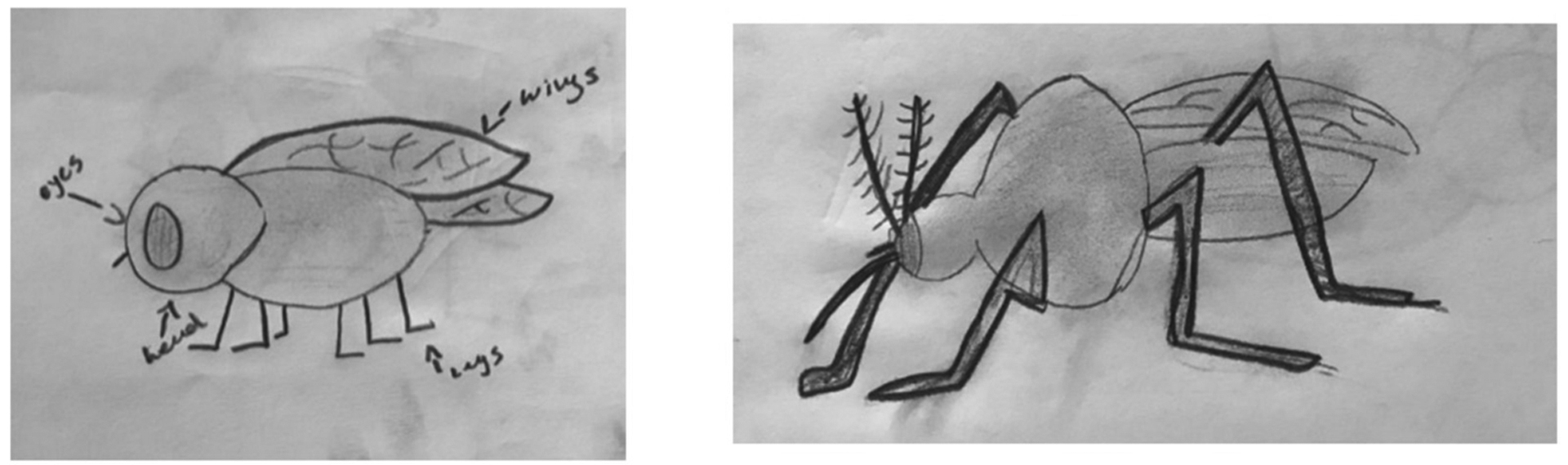
Student illustration of a mosquito.

**FIGURE 5 |:**
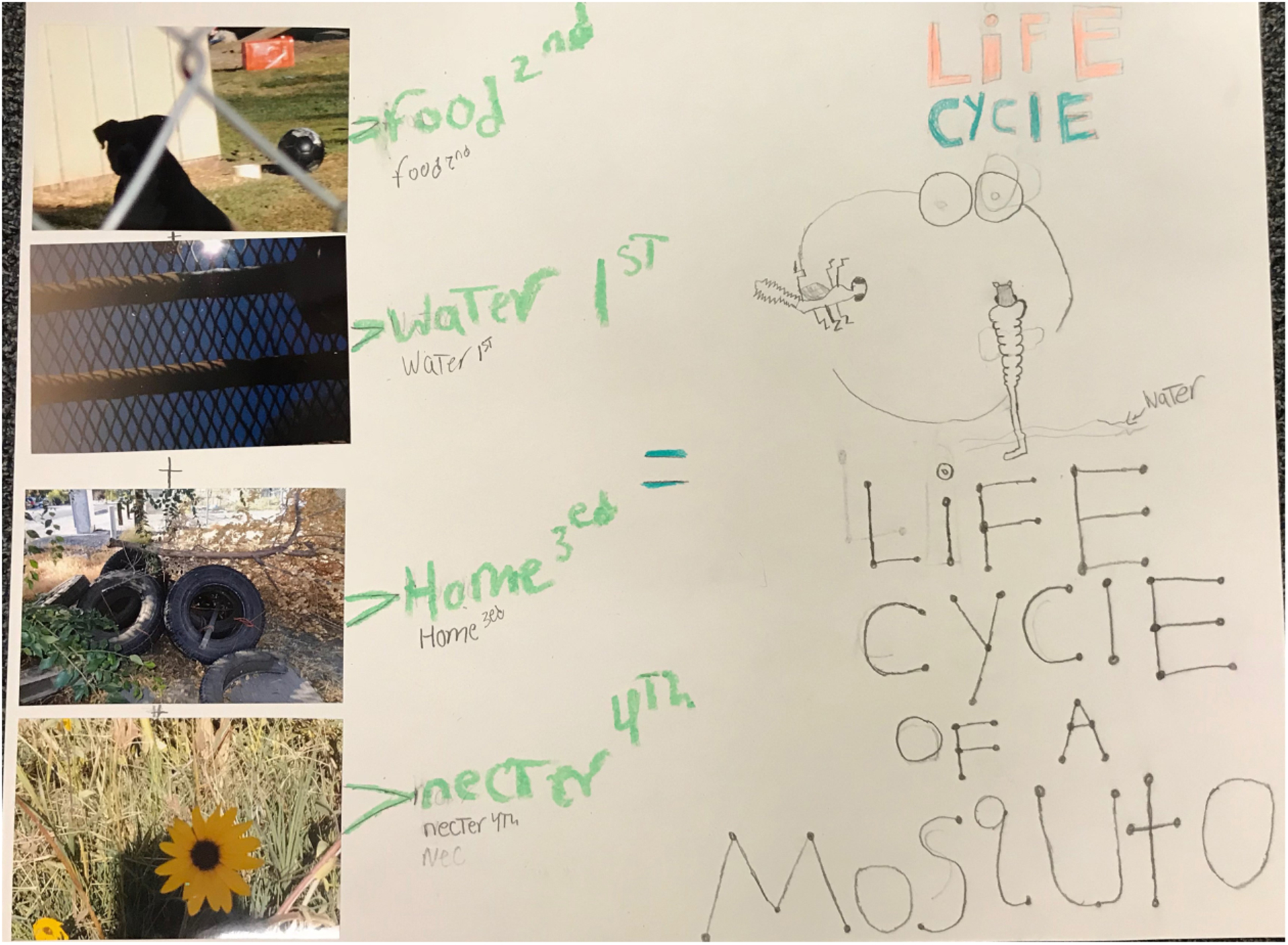
Student collage of the mosquito lifecycle.

Finally, to further illustrate the change in student depictions, we present several drawings from the students. Figure 3 shows the change in understanding of the morphology and anatomy of a mosquito, and Figure 4 further illustrates the change in the application of artistic techniques and sophistication of student drawings (see Figures 3, 4 below). To further illustrate the understanding of the connection between the students’ community and the mosquito habitat and lifecycle, we present a collage that is composed of an illustration of the mosquito lifecycle and photographs from the community where this lifecycle takes place in Figure 5 (see Figure 5 below).

Present in our analysis was evidence supporting three of the six capacities of systems thinking presented above: attendance to multiple elements and their relationships, recognition of multiple interacting causal forces, and making sense of cascading influences as a result of changes to one part of the system. Not evident was an increase in student capacity to make distinctions across scales of analysis, to understand underlying functions and structures, and to shift toward a decentralized form of sense-making about scientific processes. For example, the image featuring money was selected infrequently in both the pre- and post-image selection. A recognition of cost, an indirect social and economic implication of mosquito and disease systems, would have demonstrated a greater shift in systems thinking. This was similarly seen with the very low rates of selection for the Economist. Additionally, while selection of images related to medicine (e.g., ambulance, nurses, or vaccines) were selected more frequently after the program, student descriptions indicated they were selected because mosquitoes can make you sick. These descriptions did not extend to include greater systematic ideas surrounding healthcare cost and access – for example, that in agricultural regions students often have both more exposure to birds, livestock, and flowering plants (mosquito food) and less access to healthcare (ambulances, nurses, and vaccines), which places them at an increased risk for zoonotic diseases. And while a wider awareness of the careers that are associated with preventing mosquito-borne disease transmission were selected, there was a decline in the selection of the careers. These results are discussed further below.

## DISCUSSION

Our afterschool program expanded systems thinking through increased recognition of elements related to mosquito systems, increased awareness of interacting causal forces, and greater realization of the influence of change within a system. The capacities of systems thinking that were evident in these data, along with those that were not, have implications for practitioners as they design and implement arts and science integration to support systems thinking. Specific systems thinking capacities, such as making distinctions across scales of analysis, may require more explicit instruction and scaffolding on the part of the program designer and educator. Additionally, more research is needed to understand how to best support students in developing these systems thinking capacities.

The use of the image selection task also has implications for future research around systems thinking. We found this method to be a relatively effective way to discern student systems thinking, which did not rely heavily on either written language or longer, more formal assessments. When coupled with student descriptions explaining why images were selected, it allowed greater insight into student thinking in surprising ways. As students described their selections, they often made connections to mosquitoes through multiple aspects of the image. For example, an image of a nighttime cityscape allowed a student to demonstrate their understanding that mosquitoes are more active at night. This is interesting as focusing on one aspect of the image – the time of day, while excluding other factors (mosquitoes are less prevalent in urban areas) allows for a more nuanced understanding of how students interpret these images. Another image – a swimming pool, gave one student an opportunity to demonstrate understanding that mosquitoes need water to lay their eggs. This is particularly important as poorly maintained swimming pools allow for mosquitoes to thrive in more urban environments. This inclusion of student descriptions allowed for more open-ended solicitation of student systems thinking and understanding of scientific concepts.

We would encourage educators and practitioners to consider using images, especially photographs, to probe students for their understandings. By anchoring abstract questions about complex systems to something concrete, students can explore the images and create multiple connections (depending on the content of the picture). For example, a picture of several agricultural workers in a field may elicit multiple explanations increasing in complexity – students may focus on the person (who is full of blood), the location (mosquitoes are likely found in fields), the profession (agricultural workers are at increased risk of mosquito exposure) and the community (local communities of agricultural workers may have less access to healthcare and at the same time more exposure to mosquitoes and pesticides). We contend this exploration can serve as both a product (illustrating learning) and a process (facilitating learning).

Practitioners could also encourage students to create their own photographs or cartographic representations to further illustrate their understanding of both the arts and science concepts. Looking to one of the students’ collages (Figure 5), as an example of both a product and a process, a teacher could start with the picture of the mosquito and students could collaboratively build both the collage and the understanding over time. As the collages are completed, the dialog could move into the community by inviting students to display this artwork for parents, teachers, students, and community members to spark further discussion. This iterative, communal meaning-making could then lead to a deepened understanding of the community as a system, and how changes might be implemented.

The implementation of Buzzing for Blood described in this study reflected an attempt to address and equally value both arts and science learning outcomes and practices. This is an ongoing effort and challenge for our team as well as the STEAM movement more broadly. For example, the initial design of Buzzing for Blood was primarily led by a disease ecologist and a learning scientist, with broader advisory input from artists and arts educators. We are currently working on a next iteration of the program that focuses on synergies between cartographic arts and mosquito spatial ecology. To do this, we have begun collaborating more closely with a cartographic artist to shape both specific activity design as well as conceptual goals related to cartographic-arts learning objectives and techniques. We suggest that the nature and quality of interdisciplinary STEAM integration hinges, in part, on *who* is at the table during the learning environment design process.

The career selection task also illuminated children’s systems thinking by expanding their understanding of the wide array of careers associated with both the agricultural and healthcare sectors. Since these students find themselves at the center of these colliding worlds (with many being children of agricultural workers with limited access to healthcare and an increased exposure to mosquito-borne pathogen transmission), we are glad to have made an impact in their expanded understanding. However, there is still much work to be done.

These results illustrate the potential for arts integration to promote increased systems thinking while facilitating students to critically analyze how the West Nile Virus and mosquito morphology and ecology connects to their lives. These connections, in turn, allow them to identify risks in their own agricultural communities and aid the prevention of the West Nile Virus and other zoonotic diseases.

## Figures and Tables

**TABLE 1 |:** Weekly Buzzing for Blood curriculum.

Unit	Focal art modality	Key scientific themes	Example activities
1	Scientific illustration	Mosquito morphology	Exploring shading techniques (e.g., stippling or cross-hatching) Illustrating mosquitoes Observing mosquitoes through a microscope
2	Scientific illustration	Mosquito life cycle	Illustration techniques for depicting movement or action Observation of live mosquito larvae behaviors Illustrating larval movement and behaviors
3	Infographics	Mosquito food sources	Introduction to types and purposes of scientific infographics Science inquiry experiment about larval food concentrations under varying levels of drought Infographic construction about mosquito food sources at each life cycle stage
4	Photography	Local mosquito habitat	Exploration of photographic composition (e.g., subject placement) Neighborhood walk and photographic documentation of local mosquito habitat
5	Cartography	Mosquito spatial ecology	Example-based discussion of different types of maps Constructing neighborhood maps of local mosquito habitat
6	Multi-media / Student Choice	Cross-connections	Construction and presentation of student-led final art pieces Community art show

## Data Availability

The raw data supporting the conclusions of this article will be made available by the authors, without undue reservation.
